# Morphological Variations in Placentas among Deliveries in the Department of Obstetrics and Gynaecology in a Tertiary Care Centre: A Descriptive Cross-sectional Study

**DOI:** 10.31729/jnma.7829

**Published:** 2022-09-30

**Authors:** Iju Shrestha, Pratigyan Gautam, Sanam Khaiju

**Affiliations:** 1 Department of Anatomy, Kathmandu Medical College and Teaching Hospital, Duwakot, Bhaktapur, Nepal; 2 Department of Obstetrics and Gynaecology, Kathmandu Medical College and Teaching Hospital, Sinamangal, Kathmandu, Nepal

**Keywords:** *placenta*, *prevalence*, *umbilical cord*

## Abstract

**Introduction:**

The full term human placenta is a flattened discoidal mass with an approximately circular or oval outline. However, variations can be present in the morphology in the form of bidiscoidal shape, lobed, diffused, fenestrated or circumvallate. The objective of the study was to find out the prevalence of morphological variations in placentas among deliveries in the Department of Obstetrics and Gynaecology in a tertiary care centre.

**Methods:**

A descriptive cross-sectional study was conducted among total in the Department of Obstetrics and Gynaecology in a tertiary care centre after receiving ethical approval from the Institutional Review Committee (Reference number: 2308202105). The study was conducted between September 2021 to November 2021 and the shape of the placenta was observed and the variations present were noted. Point estimate and 95% Confidence Interval were calculated.

**Results:**

Out of 105 placentas observed, morphological variations were seen in two (1.91%) (0-4.53, 95% Confidence Interval). One (50%) was observed to be placenta succenturiata and one (50%) was triangular in shape.

**Conclusions:**

The prevalence of morphological variations in placentas was found to be higher than the studies done in similar settings.

## INTRODUCTION

The full-term human placenta is a flattened discoidal mass with an approximately circular or oval outline.^1^ In 90% of individuals a discoid or oval-shaped placenta is seen.^2^ However, variations can be present in the morphology in the form of bidiscoidal shape, lobed, diffused, fenestrated or circumvallate.^3^

Abnormal shapes are seen in 10% of women and include notched or lobed placentas, membranous placentas, and other rare variants.^2^ However, relatively less research has been done for the morphology and the variations present in its shape. So this study can be helpful in better diagnosis and management of the predisposing clinical conditions related to maternal and foetal health.^4-6^ This can also help to minimize the complications related to placenta and umbilical cord and improve maternal and fetal outcome.^7^

This study was done to find out the morphological variations among deliveries in the Department of Obstetrics and Gynaecology in a tertiary care centre.

## METHODS

This descriptive cross-sectional study was carried out in 105 placentas obtained from deliveries during the period of September 2021 to November 2021, at the Department of Obstetrics and Gynaecology of Kathmandu Medical College and Teaching Hospital after receiving ethical approval from the Institutional Review Committee (Reference number: 2308202105).

Placentas were collected from the labour room of the Department of Obstetrics and Gynaecology. After separating the baby from the umbilical cord, the specimens were placed in a bucket containing 10% formalin. Samples were then washed clean of blood and stored again in formal saline for further examination for shape of each placenta was done. The normal morphology and the variations observed were recorded. All the placentas obtained from deliveries in the study duration were included except for the damaged and torn placentas, which were excluded. The sample size was calculated by using the following formula:


$$\begin{array}{ll} \text{n} & ={\text{Z}}^{2}\times \frac{\text{p}\times \text{q}}{{\text{e}}^{\text{2}}}\\ & =1.96^{2}\times \frac{0.065\times 0.935}{0.05^{2}}\\ & =94 \end{array}$$

Where,

n= minimum required sample size
Z= 1.96 for 95% Confidence Interval (CI)
p= prevalence taken as 6.5% from educated guess
e= margin of error, 5%

Hence, the minimum required sample size was 94. However, a sample size of 105 was taken. The collected data was compiled, entered and analysed in Microsoft Excel 2013. Point estimate and 95% CI were calculated.

## RESULTS

**Figure 1:**
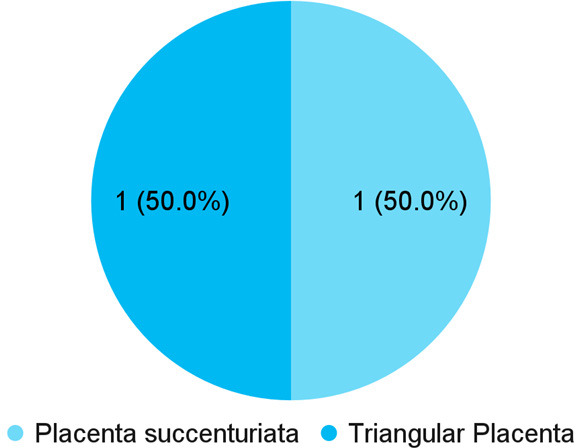
Morphological variants of placentas (n= 2).

Among 105 placentas observed, morphological variations were seen in two (1.91%) (0-4.53, 95% CI). One (50%) was placenta succenturiata and one (50%) was triangular in shape (Figure 1).

**Figure 2:**
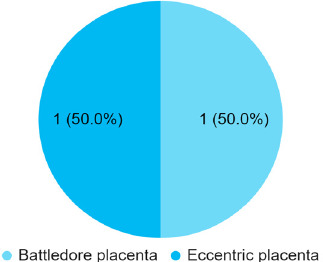
Pattern of umbilical cord attachment in placenta (n= 2).

Among these, marginal insertion umbilical cord in placenta was observed in triangular placenta whereas normal eccentric umbilical cord insertion was seen in placenta succenturiata (Figure 2).

## DISCUSSION

The full term human placenta is a flattened discoidal mass with an approximately circular or oval outline.^1^ In the present study, almost 98% of the placentas were observed with discoidal shape. This finding is in accordance with a study where it is mentioned that a discoid or oval-shaped placenta is seen in about 90% of the individuals.^2^ Similar finding of 94% circular and 6% oval shape of placenta has also been reported.^4^ Meanwhile, 48% placentas with oval and 36% with circular shape has been reported in a study.^8^

However, variations can be present in the morphology in the different forms, e.g. bidiscoidal shape, lobed, diffused, fenestrated or circumvallate.^3^ Abnormal shapes are seen in 10% of women and include notched or lobed placentas, membranous placentas, and other rare variants.^2^ In this study, one case of placenta succenturiata and a triangular placenta were observed. In this study, one case of placenta succenturiata and a triangular placenta were observed. Similar finding has been reported in a study, of one case of placenta succenturiata whereas they have reported four triangular shaped placentas.^8^ Placental abnormalities are considered as uncommon finding in obstetrics and among them the succenturiate lobe of placenta is a very rare entity.^9^ Apart from these, irregular shape of placentas has also been reported in different studies.^10,11^

The attachment of the umbilical cord was observed to be in the central portion of placenta in 104 (99.05%) placentas. In one placenta (0.95%), the cord was attached marginally. In a study conducted in Nepal, no abnormality regarding the attachment of cord, was recorded.^4^ Almost in consistency with the present study, 2% marginal attachment of the cord has been reported in a study conducted in Pakistan.^7^ Other studies have reported 7%, 8.97%, 25% marginally attached umbilical cord.^2,6,12^ These variations in placental morphology has been looked upon as being corelated to any predisposing conditions such as placenta praevia, vaginal bleeding, and premature delivery.^13^

In this present study, there were certain limitations. The occurrence of various shapes of placenta were observed, however, correlation between the morphology (shape) of placenta and any predisposing factors is yet to be studied in detail.

## CONCLUSIONS

The prevalence of morphological variants in our study was found to be higher when compared to other studies from similar settings. A sound knowledge of the abnormal morphologies may help avoiding any complications as in bilobed placenta or extra lobes being mistaken for retained placental tissues. Further studies of the shape of placenta with co-relation to predisposing conditions may also help in better diagnosis and management of the predisposing clinical conditions related to maternal and foetal health.
